# Perceptual Characterization and Analysis of Aroma Mixtures Using Gas Chromatography Recomposition-Olfactometry

**DOI:** 10.1371/journal.pone.0042693

**Published:** 2012-08-17

**Authors:** Arielle J. Johnson, Gregory D. Hirson, Susan E. Ebeler

**Affiliations:** Department of Viticulture and Enology, Agricultural and Environmental Chemistry Graduate Group, University of California Davis, Davis, California, United States of America; Alexander Flemming Biomedical Sciences Research Center, Greece

## Abstract

This paper describes the design of a new instrumental technique, Gas Chromatography Recomposition-Olfactometry (GC-R), that adapts the reconstitution technique used in flavor chemistry studies by extracting volatiles from a sample by headspace solid-phase microextraction (SPME), separating the extract on a capillary GC column, and recombining individual compounds selectively as they elute off of the column into a mixture for sensory analysis (Figure 1). Using the chromatogram of a mixture as a map, the GC-R instrument allows the operator to “cut apart" and recombine the components of the mixture at will, selecting compounds, peaks, or sections based on retention time to include or exclude in a reconstitution for sensory analysis. Selective recombination is accomplished with the installation of a Deans Switch directly in-line with the column, which directs compounds either to waste or to a cryotrap at the operator's discretion. This enables the creation of, for example, aroma reconstitutions incorporating all of the volatiles in a sample, including instrumentally undetectable compounds as well those present at concentrations below sensory thresholds, thus correcting for the “reconstitution discrepancy" sometimes noted in flavor chemistry studies. Using only flowering lavender (*Lavandula angustifola* ‘Hidcote Blue’) as a source for volatiles, we used the instrument to build mixtures of subsets of lavender volatiles in-instrument and characterized their aroma qualities with a sensory panel. We showed evidence of additive, masking, and synergistic effects in these mixtures and of “lavender' aroma character as an emergent property of specific mixtures. This was accomplished without the need for chemical standards, reductive aroma models, or calculation of Odor Activity Values, and is broadly applicable to any aroma or flavor.

**Figure 1:**
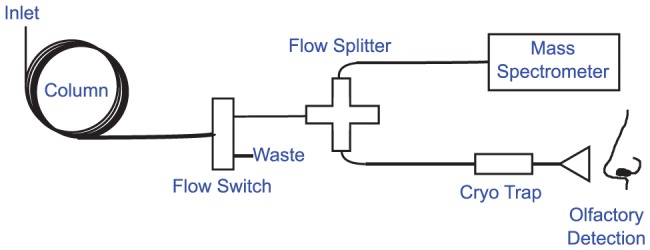
Conceptual schematic of the Gas Chromatograph Recomposition-Olfactometer (GC-R) instrument. Volatiles are extracted onto a solid phase (via solid-phase microextraction or SPME) from the headspace of a food, beverage, or other sample, in this case, lavender flowers, and initially they are separated conventionally on an analytical capillary GC column. In-line with the GC column, a pneumatic Deans Switch followed by a cold trap allows the experimenter to build a mixture of these separated volatiles that is held until the cryotrap is rapidly heated, releasing the mixture for a subject to smell at the olfactory port and evaluate.

## Introduction

Aroma plays a dominant role in the multisensory perception of flavor. It is itself a construct perceived in response to stimulation of the olfactory system by volatile chemicals and mixtures thereof, with mixtures being commonly encountered in everyday life in the form of food, wine, plants, perfume, etc. While our understanding of the neurobiological and psychological mechanisms that translate volatiles into aroma perceptions has advanced significantly in recent years [1], [2], analytical approaches for characterizing the perception of these aroma mixtures are still limited. The relationship between chemical composition of a mixture of volatiles and its perceived aroma or flavor is complex and difficult to predict on the basis of chemical data or simple sensory data alone.

Analytical chemistry approaches for characterizing aromas or flavors typically rely on separation-based chromatographic methods that quantify the aroma strength of individual compounds in a mixture, reflected as either the concentration present in the mixture divided by a measured sensory threshold concentration (Odor Activity Value, OAV) [3], [4] or the number of N-fold dilutions required to suppress detectability of a compound when analyzed by gas chromatography with a human subject acting as an olfactory detector (GC-Olfactometry or GC-O; CHARM; or Aroma Extract Dilution Analysis) [5]–[7]. Reconstitution and omission experiments evaluate the role of specific compounds in the perceived aroma of a mixture, whereby a blend of compounds hypothesized to be detectable in a food, beverage, or other sample by OAV is mixed from chemical standards, and compared to similar mixtures prepared by omitting one of these compounds at a time [7]. If a difference is detectable in the “whole" mix versus a “whole-minus-one-compound" mix, that particular compound is considered important to the aroma of the sample.

Knowledge from other disciplines studying aroma, such as sensory psychophysics, cognitive psychology, and molecular neurobiology, suggests limitations of these methodologies. Chromatographic techniques only assess the aroma quality of individual compounds, rather than mixtures of compounds. However, the aroma of a mixture is frequently perceptually distinct from that of its individual components [8], [9] and may have qualities not found in any of these components [10]. The mixing-dependent nature of aroma quality is evidenced by the relative lack of aroma impact compounds, or those compounds that are singularly responsible for the overall aroma impression of a food or beverage. On the other hand, omission experiments rely on an assumption that all sensorially important compounds have been correctly identified and quantified and that any compound occurring at a concentration below its putative sensory threshold is not important to the overall aroma. Recently published results suggest that this is not the case [11]. Despite having identical concentration profiles of supra-threshold odorants, the aroma of a reconstitution sometimes still smells different from the original mixture [12], a phenomenon referred to as “reconstitution discrepancy" [13]. Some recent omission experiments have included sub-threshold components in the reconstitution [13], but this is not a universal practice, and can greatly complicate and enlarge the experimental design.

We propose here a novel platform for the analytical characterization of aroma and flavor perception that incorporates and merges aspects of the previously described techniques and knowledge from other related disciplines. We describe a series of non-reductive, in-instrument recombination and omission experiments using a Gas Chromatograph modified with a switch and then a cold trap in-line between the capillary column and the chemical and olfactory detectors to characterize the aroma of lavender (*Lavandula angustifola* ‘Hidcote Blue’). The volatile chemical composition of lavender, a potently aromatic herb with numerous culinary, cosmetic, and fragrance uses, has previously been characterized [14], but there are no lavender impact compounds currently identified. This suggests that “lavender" aroma character arises from the perception of a mixture of volatiles rather than a single molecule, making this an ideal mixture for evaluation of perceptual interactions using our gas chromatography recomposition-olfactometry GC-R) approach.

## Materials and Methods

### Instrument

**Figure 2:**
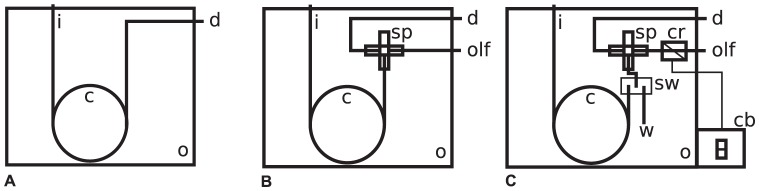
Schematic of (a) standard GC-MS; (b) GC-MS with splitter at end of column for olfactometry; and (c) Gas Chromatograph-Recomposition-Olfactometer or GC-R with Deans switch, splitter, cryogenic trap and olfactory port. Abbreviations: i-inlet; c-column; d-detector; o-oven; olf-olfactometry port; sp-splitter; sw-Deans switch 1; w-waste; cr-cryogenic trap; and cb-switch 2 on control box.

An Agilent model 6890 gas chromatograph/5972 mass spectral detector (GC-MSD) was modified with the addition of a Deans switch apparatus (Agilent Technologies, Santa Clara, CA), an auxiliary pressure controller (EPC, Agilent) to control flow through the Deans switch, a splitter (Gerstel), a cryotrap (Micro Cryo-trap and model 971 controller, Scientific Instrument Services, Ringoes NJ) and an olfactometry port (ODP-2, Gerstel, Linthicum, MD). A schematic showing modifications from a standard GC-MS (Figure 2a), to a GC-O instrument (Figure 2c), to the GC-R Gas Chromatograph is shown (Figure 2c). Deactivated fused silica was used for all transfer lines. The transfer line from the Deans switch to the splitter was 4 m. The dimensions of the transfer line from the splitter to the MSD was 1 m × 0.15 mm; the dimensions of the transfer line from the splitter to the olfactory port was 1 m × 0.25 mm resulting in a 1.86∶1 split ratio between the olfactory port and MSD.

### Sampling and Chromatographic Conditions

Lavender (*Lavandula angustifola* ‘Hidcote Blue’) flowers (0.50 g) were weighed and placed in a 20 mL amber glass headspace vial and sealed with a crimp cap with a PTFE-faced silicone septum (Supelco, St. Louis, MO). A Solid Phase Microextraction fiber (2 cm length, 50/30 um divinylbenzene/carboxen/polydimethylsiloxane coating, Supelco) was used for extraction. The fiber was exposed to the headspace of the vial for 30 minutes at room temperature, then withdrawn and immediately desorbed in the GC inlet. Chromatographic conditions were adapted from [14]. Separation was performed using a 30 m×25 mm i.d. ×0.25 um film thickness DB-5MS column (J&W, Folsom, CA). The inlet was maintained at 240°C in splitless mode. Helium was used as the carrier gas and was held at constant pressure at 15.5 psi. The auxiliary pressure controller was maintained at 3.4 psi. The SPME assembly was introduced manually into the inlet and allowed to desorb for a total of 10 minutes. The oven was held at 60°C for 3 minutes, then ramped to 150°C at a rate of 3°C/min, then ramped to 325 at a rate of 30°C/min and held for 1 min for a total runtime of 40 minutes. The olfactory port transfer line was maintained at 100°C and the MSD transfer line was maintained at 260°C. After a 0.5 min solvent delay, the mass spectrometer scanned from *m/z* 50–230. With the Deans switch set in the “off" position, the flow is directed to the splitter, MSD, cold trap, and ODP. When set to the “on" position, the flow is directed to waste. The switch is programmed in the “runtime" tab of the Enhanced Chemstation Software (Hewlett Packard, version B.01.00) to direct the flow over the course of the runtime as desired by the operator.

### Sensory Conditions

**Figure 3:**
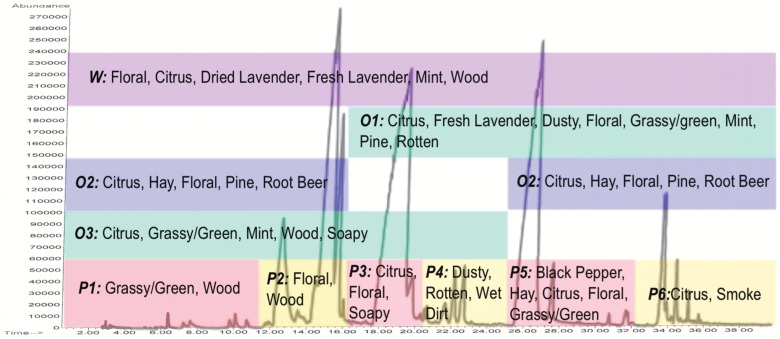
Top aroma descriptors for mixtures of sections of the lavender chromatogram by cut time and chromatogram composition. Abbreviations correspond to Experimental Conditions described in Table 1. As chemical complexity and number of components per mixture approaches the makeup of the whole chromatogram (W) mixture, there is evidence of perceptual additivity as increasing cross-utilization of terms from simpler mixtures, masking as reduced use of dominant terms for simpler (P1–P6) mixtures, and synergistic effects as new complex or composite terms like “fresh lavender" become important.

Based on retention time, the Deans Switch sends specific packets of volatiles to the cryotrap. Here we used one of ten programs (W, O1–O3, P1–P6; see Figure 3, Table 1) where at the conclusion of the separation run, the cold trap was heated and the mixture was sniffed and described by a sensory panelist. The W condition, analogous to a full aroma reconstitute, contains all the volatiles of lavender, with conditions O1–O3 and P1–P6 omitting groups of these volatiles for descriptive comparison to the aroma of the W sample and to lavender flowers.

**Table 1:** Experimental GC-O conditions and aroma descriptors for mixtures of volatiles from the lavender chromatograms.

Experimental Condition	Abbreviation	Chromatogram Sections Included in Mixture	Top Descriptors
Whole Chromatogram	W	0–40 minutes	Floral, citrus, dried lavender, fresh lavender, mint, wood
^1^Omission 1	O1	16–40 minutes	Citrus, fresh lavender, dusty, floral, grassy/green, mint, pine, rotten
^1^Omission 2	O2	0–16+25–40 minutes	Citrus, haylike, floral, pine, root beer
^1^Omission 3	O3	0–25 minutes	Citrus, grassy/green, mint, wood, soapy
^2^Perceptual Interaction 1	P1	0–11 minutes	Grassy/green, wood
^2^Perceptual Interaction 2	P2	11–16 minutes	Floral, wood
^2^Perceptual Interaction 3	P3	16–20.5 minutes	Citrus, floral, soapy
^2^Perceptual Interaction 4	P4	20.5–25 minutes	Dusty, rotten, wet dirt
^2^Perceptual Interaction 5	P5	25–32 minutes	Black pepper, haylike, citrus, floral, grassy/green
^2^Perceptual Interaction 6	P6	32–40 minutes	Citrus, smoke
Lavender Flowers Reference	Reference	Not separated; whole lavender flowers	Citrus, floral, fresh lavender, mint, wood, hay, dried lavender, grassy/green

Three panelists (Females, ages 28–45 with previous sensory experience) smelled each of the ten mixtures in triplicate and generated terms to describe the perceived odor. Before smelling each mixture, each panelist first smelled and described a standard of lavender flowers, picked at the same time as the flowers used for SPME sampling, and also rated how well the sample mixture represented the aroma of the standard on a scale of 0–10.

### Ethics Statement

Use of human subjects for this study was reviewed by the University of California, Davis Institutional Review Board and was granted exempt status (Category 6).

### Data Analysis

The terms used to describe the ten mixtures were tabulated by frequency of use. The descriptors used most often for each mixture, in a mixture-by-descriptor data matrix, were analyzed with a correspondence analysis to identify latent trends in similarity and difference in the multidimensional set. A three-way Analysis of Variance (ANOVA) with all two-way interactions was performed; rated representativeness of each mixture was compared to a fresh lavender standard as the response factor and panelist, mixture, and replicate were the main effects. A Tukey's Honest Significant Difference multiple comparisons test (HSD) was performed on the representativeness ratings. The R statistical computing package was used for all statistical analyses (http://www.r-project.org/).

## Results and Discussion

We modified a GC-MS to allow for the in-instrument preparation of volatile mixtures containing precise sections from a chromatogram, up to and including the entire volatile fraction and allowing for aroma characterization of the aroma of one or a few of the volatiles in a complex mixture (Figure 1). Compounds were introduced into the inlet of the modified GC-MS and separated on the analytical column. At the end of the column, the flow of carrier gas and analytes encountered a first switch, a commercially available Deans switch, that was set to direct the flow either towards the splitter or towards waste (here waste was vented to the oven). The splitter subsequently split the flow to both a mass spectrometer (MS) detector and to an olfactory port. Along the transfer line to the olfactory port was a trap controlled by a second switch at the control box; the switch allowed the trap to be cooled with liquid carbon dioxide or heated so that the eluant was either held within the trap (*i.e.*, cryotrapped) or released to the olfactory port. By programming the switches to cryotrap or exclude selected peaks or peak regions (Table 1) two types of experiments were performed. In perceptual interaction experiments, all of the chromatogram except for a small section of peaks was cut away, and the section of interest was assessed at the olfactory port as a mixture. In omission experiments, small groups of peaks (or individual peaks) were cut away and the rest of the compounds in the chromatogram were smelled as a mixture. See Figures S1 and S2 for examples of these chromatograms.

Using our new approach, ten aroma mixtures (Table 1, Figure 3) were created in-instrument directly from the headspace-extracted volatiles of flowering lavender. “Fresh Lavender" and “Dried Lavender" were both predominant descriptors for the “Whole Volatile" recombination mixture W. Of the more chemically complex omission mixtures O1–O3, only O1, which incorporated the section of volatiles eluting from 16–40 min of the lavender chromatogram and omitted volatiles eluting between 0–16 min, was described as having “fresh lavender" properties. O1 overlapped with O2 from 25–40 min and with O3 from 16–25 minutes and incorporated the perceptual mixtures P3–P6, however, none of these other omission or perceptual mixtures had fresh or dried lavender among their commonly used descriptors. This suggests that there are two subsets of compounds, the first eluting between 16–25 min and the other eluting between 25–40 min, that are each necessary for the perception of “lavender character" but are not alone sufficient for inducing this perception without some mixing with compounds in the other elution group. These results also suggest that “lavender character" is an emergent perceptual property arising from the mixing of these volatiles or some subset thereof.

**Figure 4:**
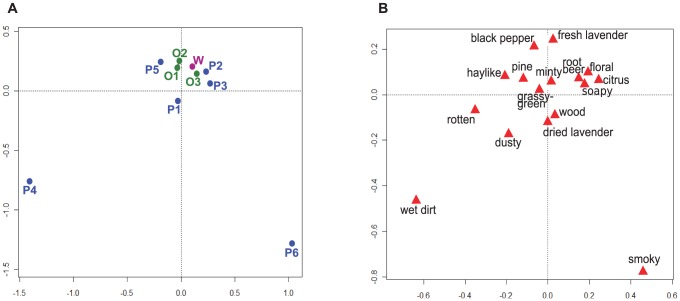
Correspondence Analysis of (A) lavender volatile mixtures; and (B) lavender volatile mixture descriptors. Abbreviations for mixtures correspond to those in Table 1. Terms generated by the panelists to describe the perceived odor of from each Experimental Condition described in Table 1 were tabulated by frequency of use and used for the Correspondence Analysis. 30.57% of variance explained by dimension 1 (x), 22.84% of variance explained by dimension 2 (y).

We performed a Correspondence Analysis on the descriptors-by-mixtures data matrix to compare dimensionally-reduced latent trends in the sensory profiles of the mixtures to the differences evident in top descriptors for each mixture (Figure 4). Correspondence Analysis separates dissimilar categories in space; mixtures and sensory descriptors spaced closely together share more similarities than those spaced further apart. This plot shows that, generally, removing more volatiles results in greater dissimilarity between a given mixture and the all-volatiles-included mixture W. The relatively tight clustering of W and omission mixtures O1–O3 in the Correspondence Analysis reflects the sensory similarity of these mixtures; perceptual mixtures P2 and P3 also cluster nearby, reflecting some of the overlapping characteristics of these mixtures (Figure 4).

The location of mixture W in the center of the main cluster in the Correspondence Analysis, suggests its aroma was perceived, in part, as a sensory average of some of the less-complex mixtures. However, a truly averaged perceptual character would be in the center of the plot; the fact that mixture W is offset from the geometric center implies that the mixing-dependent interactive effects of the lavender volatiles perceived in mixture W play a noticeable role in affecting its overall aroma character. Mixture W shares many similar descriptors (Table 1) with O1–O3 and P2 and P3, but all of these except O1 lack a dominant lavender character.

Mixtures P1 and P5 are close to the central cluster but are approximately equi-distant in space from mixture W. This reflects some of the similarities in the descriptors that P1 and P5 share with mixture W, but also reflects the domination of the aromas of these mixtures by either a unique character (“black pepper") in the case of P5, or the relative simplicity of the aroma in the case of P1 (Figure 4a). The comparative distancing of mixtures P4 and P6 from the other mixtures reflects the relative uniqueness of their aroma descriptors.

Locations of descriptors suggest that along the first (x) dimension of Figure 4b, there is a distinction between fresher, more “sweet" and flower-associated terms on the right side and earthier, heavier aroma terms on the left. Borrowing more qualitative terms from the tradition of perfumery (which at its essence is the craft of observing and optimizing the perceptual effects of mixing volatiles), we observe a rough progression, from left to right along the x-axis, of base, middle, and top-note [15] related terms. Along the second (y) dimension the separation is dominated by the marked difference of P4 and P6 from each other and from the rest of the mixtures, and correspondingly by their unique descriptors “wet dirt" and “smoky" in Figure 4b. Generally, the terms on the other arm of the y-dimension tend to be shared by multiple mixtures, or reflect more composite aroma characteristics.

While sample P1 appears to be the closest to the central or average sample in this set, it is clearly separated from the cluster centered around mixture W along the third (z) dimension (Figure S3). The third dimension also further separates mixture P5 from the central W-associated cluster and increases the distinction between “grassy/green"-“woody" descriptors on one side and “dried lavender"-“black pepper" descriptors on the other. Importantly, the Correspondence Analysis, while unable to describe absolute differences, provides valuable information not only on the sources of variation in the complex sensory data but also on the interrelationships of the mixtures and their sensory properties.

The method used to create an extract of volatile compounds can alter the perceived aroma of that extract and failure to obtain a representative sample can lead to unreliable conclusions about the composition of the aroma active components [16]–[20]. While many extraction methods have been employed in order to produce an aroma extract [19]–[21], the creation of a representative aroma can be very difficult for complex matrices [20], [22], and the sensory representativeness of this extract is not always evaluated. Here, the aroma of the SPME extracts of lavender corresponded closely to the original product (Table 1). Similar representative aroma samples have been obtained using SPME to sample “baked potato" aroma [23]. Importantly, the GC-R approach provides a rapid, easy, and effective tool to assess the representativeness of an extract regardless of the extraction method employed, such as in cases where SPME coatings may not be able to produce an appropriate extract [24].

**Figure 5:**
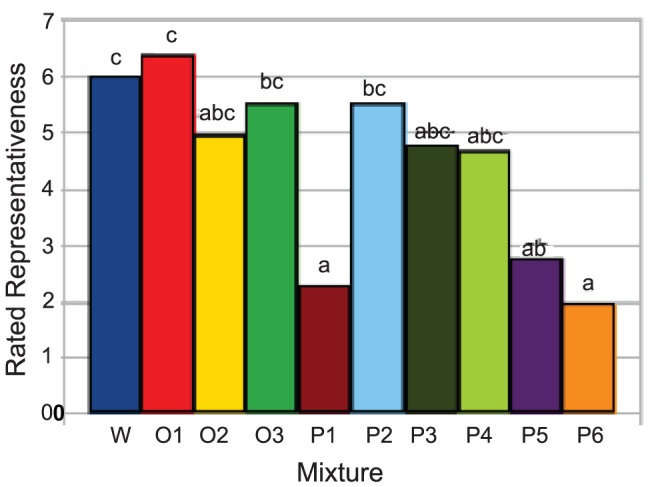
The rated representativeness of the aroma of samples W, O1–O3, and P1–P6 as compared by panelists to the aroma of whole flowering lavender. Letters a, b, c refer to the mixture's Significant Difference from each other- if two samples do not share a letter, they are significantly different. Samples P1, P5, and P6 are significantly less representative of the aroma of flowering lavender than sample W, which incorporates all the volatiles in flowering lavender.

Since the SPME extraction produced an aroma mixture representative of lavender, it was possible to perform omission and interaction experiments based on a starting point nearly identical to the intact lavender sample, eliminating “reconstitution discrepancy" [13]. Comparing the aroma of the GC-R mixtures in this study to the aroma of whole lavender flowers, panelists found that mixtures P1, P5, and P6 were significantly less representative (Figure 5) of the aroma of the whole flowers than mixtures W, O1–O3 and P2–P4. These samples also tended to have either fewer commonly used descriptors or descriptors not found for other mixtures (such as “black pepper" for P5 and “smoke" for P6; Table 1).

In this experimental design, mixtures of compounds were omitted to assess the resulting aroma. Cut times were chosen to include chemically similar compounds in the same mixture, for example, monoterpene acetate esters in mixture P5 and sesquiterpenes in mixture P6. However, the omitted compounds/fractions in a theoretical GC-R experiment need not be contiguous. It is possible, for example, to remove every other chromatographic peak, to remove only the 3rd and 17th peak, etc. while trapping and evaluating the remaining components. The apparatus could additionally be used to perform single omission experiments, where compounds are omitted one at a time to screen for potential impact odorants, or perceptual interaction experiments where only 2 or 3 peaks are included in the mixture. The flexibility in the compounds that can be removed and assessed is only limited by the rapid switching time of the Deans switch. By using a Mass Spectrometric detector, compounds in the sample can be identified (Table S1) however, an obvious advantage of performing an omission experiment in this manner is that the compounds need not be identifiable or available to perform the experiment. Reconstitution experiments often require the experimenter to perform lengthy and labor-intensive syntheses to prepare a component for the reconstitution model [12] only to find that the component can be omitted with no change in the overall aroma of the solution. Furthermore, there is always some fraction of the total compounds identified that are not included in the reconstitution because they are deemed to have a concentration too low to have an effect on the overall aroma. However, compounds with low odor activity values often still have a considerable effect on the overall aroma of the mixture [11], [25], [26]. With this instrument there is no simplified reconstitute - the omission experiment is performed on the entire sample.

While compounds with low OAVs may be important to the aroma of the mixtures, the opposite case can also occur, and the sensitivity of the human nose is frequently orders of magnitude greater than an instrumental detector. As a result, the nose may detect an aroma where there is no peak on a chromatogram [17]. Particularly as compared to reconstitution studies, this is another distinct advantage of the GC-R approach since even compounds not detected by the detector (MS, FID) will be included in the aroma sample as it is assessed by a subject at the olfactometry port. Traditionally, full separation of volatile compounds on the chromatographic column is necessary in order to meaningfully describe the aroma character of the eluant by GC-O since it simplifies the recognition task for the assessor [21]. However, it is more often the case that a complex mixture of aroma compounds is responsible for the overall aroma of a food or beverage. In addition, a mixture of two or more odorants can frequently lead to an aroma that is not similar to any of its individual components [10], [27]. Using a GC-R technique, any of these interactions can readily be investigated; and all that is necessary to characterize any type of aroma interaction is a sample of the food, beverage, flower, etc. of interest. Compounds detectable by GC-O but not GC-MS, compounds below putative aroma thresholds, compounds at levels that cannot be quantified, and compounds not commercially available or easily synthesized can all be perceptually analyzed if they are found in one or more aromatic samples available to the researcher.

## Conclusions

The perception of aroma and flavor has often been approached as a problem of many individual parts, with chemistry, neurobiology, sensory science, psychology, and other disciplines focused on answering questions about some aspect of the relationship between stimulus (a flower, a glass of wine, a plate of food), response (perceived flavor, liking or disliking, intake and satiety), or the pathway between the two (genetics, receptor binding, transduction, translation to cortical neurons). This has yielded a great deal of information about those individual parts, but not a well-developed understanding of how they work together for complex, everyday stimuli and activities like eating and drinking. The need for a holistic approach to address this has been identified previously [28], *i.e.*, a praxis which would bring together knowledge and research techniques from these diverse, often isolated, but orthogonally-related scientific fields, and would include expertise or information from applied, non-analytical fields with a well-developed shared intuition about the nature of aroma and flavor in practice, such as cuisine and perfumery. While the described approach of in-instrument gas chromatography recombination-olfactometry has its roots in a traditional coupling of analytical chemistry and sensory science, it is highly informed by this multidisciplinary understanding of aroma and flavor and allows for the analysis of previously uncharacterized emergent perceptual properties of complex mixture interaction effects in everyday smell and flavor situations.

## Supporting Information

Figure S1 **The chromatogram of mixture O2.** Compounds eluting between 16 and 25 minutes were vented to waste by the Deans Switch and were consequently excluded from the smelled mixture and not sent to the mass spectrometer.
(TIF)

Figure S2 **The chromatogram of mixture P5.** Compounds eluting between 0 and 25 minutes and 32 and 40 minutes were vented to waste by the Deans Switch and were consequently excluded from the smelled mixture and not sent to the mass spectrometer.
(TIF)

Figure S3 **Alternate views of correspondence analysis (** **Figure 4** **) incorporating the first 3 dimensions of variation.** 30.57% of variance explained by dimension 1 (x), 22.84% of variance explained by dimension 2 (y), 14.03% of variance explained by dimension 3 (z).
(EPS)

Table S1 **Tentative identification of lavender volatile compounds.** Volatiles were identified by matching their mass spectra to the NIST 05 Mass Spectral Library (National Institute of Standards and Technology, Gaithersberg, MD) and to chemical standards, as noted. The table is divided by cut time for perceptual mixtures P1–P6.
(DOC)
