# Supplementary figures and images for: Perceptual Characterization and Analysis of Aroma Mixtures Using Gas Chromatography Recomposition-Olfactometry
Source: PLoS One. 2012 Aug 17;7(8):e42693. doi: 10.1371/journal.pone.0042693 (PMC3422294; doi:10.1371/journal.pone.0042693)

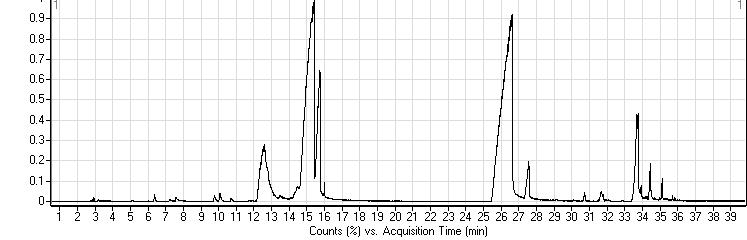

Supplement: Figure S1 — The chromatogram of mixture O2. Compounds eluting between 16 and 25 minutes were vented to waste by the Deans Switch and were consequently excluded from the smelled mixture and not sent to the mass spectrometer. (TIF) [file pone.0042693.s001.tif]

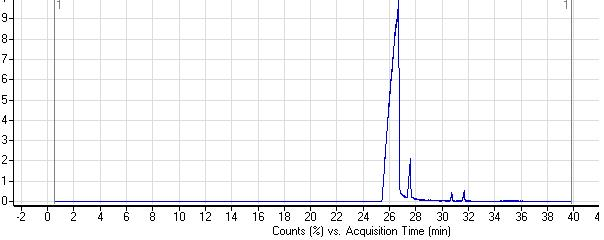

Supplement: Figure S2 — The chromatogram of mixture P5. Compounds eluting between 0 and 25 minutes and 32 and 40 minutes were vented to waste by the Deans Switch and were consequently excluded from the smelled mixture and not sent to the mass spectrometer. (TIF) [file pone.0042693.s002.tif]
