# Supplementary material for: Perceptual Characterization and Analysis of Aroma Mixtures Using Gas Chromatography Recomposition-Olfactometry
Source: PLoS One. 2012 Aug 17;7(8):e42693. doi: 10.1371/journal.pone.0042693 (PMC3422294; doi:10.1371/journal.pone.0042693)
Supplement: Table S1 — Tentative identification of lavender volatile compounds. Volatiles were identified by matching their mass spectra to the NIST 05 Mass Spectral Library (National Institute of Standards and Technology, Gaithersberg, MD) and to chemical standards, as noted. The table is divided by cut time for perceptual mixtures P1–P6. (DOC) [file pone.0042693.s004.doc]

**Supplementary Information**

**Table S1**. Tentative identification of lavender volatile compounds. Volatiles were identified by matching their mass spectra to the NIST 05 Mass Spectral Library (National Institute of Standards and Technology, Gaithersberg, MD) and to chemical standards, as noted. The table is divided by cut time for perceptual mixtures P1-P6.

| **Retention Time** | **Compound** | **CAS Number** | **Retention Index** | **Identification** | **Perceptual Mixture Group** |
| --- | --- | --- | --- | --- | --- |
| 7.23 | 3-hexen-1-ol | 544-12-7 | 893 | standard | P1 |
| 7.64 | 1-hexanol | 111-27-3 | 900 | standard | P1 |
| 9.82 | alpha-thujene | 2867-05-2 | 940 | library | P1 |
| 10.15 | alpha-pinene | 80-56-8 | 946 | standard | P1 |
| 10.79 | camphene | 79-92-5 | 958 | standard | P1 |
| 11.83 | sabinene | 3387-41-5 | 978 | library | P2 |
| 12.00 | beta-pinene | 127-91-3 | 982 | standard | P2 |
| 12.45 | 3-octanone | 106-68-3 | 990 | standard | P2 |
| 12.57 | myrcene | 123-35-3 | 993 | standard | P2 |
| 12.72 | not identified |  | 996 |  | P2 |
| 13.15 | alpha-phellandrene | 99-83-2 | 1004 | library | P2 |
| 13.50 | hexyl acetate | 142-92-7 | 1011 | standard | P2 |
| 13.23 | not identified |  | 1006 |  | P2 |
| 13.65 | 3-carene | 13466-78-9 | 1014 | standard | P2 |
| 14.52 | not identified^1^ |  | 1032 |  | P2 |
| 14.67 | eucalyptol | 470-82-6 | 1035 | standard | P2 |
| 15.41 | trans-beta-ocimene | 3779-61-1 | 1050 | library | P2 |
| 15.79 | cis-beta-ocimene | 3338-55-4 | 1058 | library | P2 |
| 16.06 | gamma-terpinene | 99-85-4 | 1064 | library | P3 |
| 16.44 | beta-terpineol | 138-87-4 | 1072 | library | P3 |
| 17.34 | terpinolene | 586-62-9 | 1091 | standard | P3 |
| 18.90 | linalool | 78-70-6 | 1125 | standard | P3 |
| 18.62 | octen-1-ol acetate | 32717-31-0 | 1119 | standard | P3 |
| 19.04 | 3-octylacetate | 4864-61-3 | 1128 | library | P3 |
| 19.64 | allo-ocimene | 7216-56-0 | 1142 | library | P3 |
| 20.21 | lavandulol | 507-70-0 | 1155 | library | P3 |
| 20.43 | camphor | 464-49-3 | 1160 | standard | P3 |
| 22.08 | terpinen-4-ol | 562-74-3 | 1199 | standard | P4 |
| 22.66 | hexyl butyrate | 2639-63-6 | 1212 | library | P4 |
| 23.20 | alpha-terpineol | 98-55-5 | 1225 | standard | P4 |
| 24.34 | bornyl formate | 7492-41-3 | 1253 | library | P4 |
| 26.53 | linalyl acetate | 115-95-7 | 1309 | library | P5 |
| 27.28 | isobornyl acetate | 125-12-2 | 1328 | library | P5 |
| 27.42 | lavandulyl acetate | 25905-14-0 | 1332 | library | P5 |
| 27.71 | geranyl acetate | 105-87-3 | 1340 | library | P5 |
| 30.63 | neryl acetate | 141-12-8 | 1420 | library | P5 |
| 31.67 | Not identified ^2^ |  | 1449 |  | P5 |
| 32.85 | alpha-bergamotene | 17699-05-7 | 1484 | library | P6 |
| 33.65 | alpha-santalene | 512-61-8 | 1507 | library | P6 |
| 33.78 | beta-caryophyllene | 87-44-5 | 1511 | standard | P6 |
| 34.48 | beta-farnesene | 77129-48-7 | 1532 | standard | P6 |
| 34.99 | alpha-bisabolene | 17627-44-0 | 1547 | library | P6 |
| 35.16 | germacrene D | 37839-63-7 | 1553 | library | P6 |
| 35.73 | alpha-amorphene | 483-75-0 | 1570 | library | P6 |
| ^1^possible terpene | |  |  |  |  |
| ^2^possible acetate ester | |  |  |  |  |
